# Editorial

**Published:** 1870-03

**Authors:** 


### Editorial.

ALUMNI ASSOCIATION OF CHICAGO MEDICAL COLLEGE.—The members of this Association will hold their annual meeting in the Hall of the Chicago Medical College, on Tuesday, March 22d, 1870. Meetings will be held for the reception of reports, papers, and the transaction of business, at 11 o'clock A.M., and at 4 P.M.

A social entertainment may be expected in the evening.

It is earnestly desired that as many of the Alumni, from all parts of the country, as possible, will attend. The commencement exercises of the college will take place the same day, at 2½ o'clock P.M.

COLLEGE COMMENCEMENT.—The public commencement exercises of the Chicago Medical College, will be held in the College Hall, on Monday and Tuesday, March 21–22, 1870.

The exercises on Monday will commence at 2 o'clock P.M., and consist in the public examination of the candidates for graduation and the reading of thesis. On Tuesday, the exercises will commence at 2½ P.M., and will consist in the distribution of certificates to the undergraduates, the award of premiums for the best thesis, conferring the degrees, by Prof. Haven,

President of the North-Western University, response on the part of the class, and valedictory address by Prof. H. A. Johnson. The medical profession and the public generally are invited to attend on both days.

---

Spring and Summer Medical Instruction.—The regular summer course of instruction, in the Chicago Medical College, will commence on the first Monday in April and continue until the first of July. The course will consist of from one to two hours clinical instruction in the hospitals, and two lectures and examinations in the college each day. The instruction will be given by the faculty of the College and their assistants. It is supplementary to the regular winter term, and is free to all the matriculants of the College.

---

Medical College Convention.—We have learned that delegates to the Convention to be held in Washington, on Friday preceding the next meeting of the American Medical Association, have been appointed by many of the older and more influential schools in the country, both North and South. We can see no good reason why every medical college cannot be represented on that occasion. The schools in New York and Philadelphia have it entirely in their power to determine whether our entire college system of education shall be placed at once on a reasonable and proper basis or not. The schools in the West, and the profession generally, are willing to sustain fully the essential features of the plan adopted by the Convention of 1866. Will the colleges and universities in the Eastern cities longer let their rivalries deter them from that open concert of action which the honor and interests of the profession require?

The coming Convention will be regarded, very generally, as fully settling the question whether the medical colleges of this country can be induced to take any important steps in the work of improving our system of medical education, in concert, or not.

---

Chicago Medical Society.—The regular meetings of this

Society have been moderately well attended during the past two months, and the cases presented have been interesting, and the discussions profitable. At a recent meeting, Dr. E. A. Bogue, one of the Surgeons to the County Hospital, presented a pathological specimen consisting of the bones of the fore-arm, which had been previously amputated. The patient had died of pyemia.

The amputated end of the bone presented a partially necrosed appearance, with gangrenous spots in the pulp or medullary matter, extending some distance into the shaft of the bone. It was remarked that such appearance of gangrene had been observed in most cases in which proper examinations death from pyemia had been made.

Dr. Bogue claimed that there were two diseases very generally confounded under the name of pyemia, or septicemia. The one was derived from true *purulent* infection, and the other from putrid infection. The term *pyemia* should be restricted to the first, and that of septicemia to the second. The first he had found to be uniformly fatal, under every variety of treatment, and he thought those cases reported from time to time in the medical periodicals, as cases of pyemia successfully treated by sulphites, or other remedial agents, were not pyemia proper.

It might be difficult to draw the differential diagnosis between the cases of purulent and putrid infection. But he thought the first commenced much more insidiously, and when connected with a wound, the surface of the wound always presents a peculiarly pale and flabby appearance for twelve to twenty-four hours before the general symptoms were observable. The patient was more passive and indifferent than in septicemia; the chills and sweats more generally repeated from day to day; the progress of the case slower, and more steadily downward until the fatal result was reached. Another peculiarity was that in pyemia re-amputation or excision of the wound after the constitutional symptoms had appeared, was productive of no benefit; while in septicemia from putrid and gangrenous wounds, removal of the injured part is often followed by a rapid improvement. The case elicited an interesting discussion, which was resumed at a subsequent meeting of the Society; but no important facts or modes of treatment were elicited, not already well known to the profession.

---

MINNESOTA STATE MEDICAL SOCIETY.—The Second Annual Meeting of the Minnesota State Medical Society was held at St. Paul, February 2d, 1870.

The following were elected officers for the ensuing year:—*President*—Samuel Willey, M.D., of St. Paul; *Vice-Presidents*—N. B. Hall, M.D., of Minneapolis, W. H. H. Richards, M.D., of Winona, and Solomon Blood, M.D., of Ottawa; *Recording Secretary*—E. H. Smith, M.D., of St. Paul; *Corresponding Secretary*—C. H. Adams, M.D., of Hastings; *Treasurer*—S. B. Sheardown, M.D., of Stockton. We hope the Secretary will send us a full account of the proceedings.

---

CHANGES IN MEDICAL JOURNALS.—*The Western Journal of Medicine*, recently published at Indianapolis, Ind., has been discontinued, or rather superceded by a monthly journal, called the "*American Practitioner*," published at Louisville, Ky. The journal in its new form and location is edited by Prof. D. W. Yandell and Theophilus Parvin, and promises to be one of our best monthly medical periodicals.

---

DIPLOMA VENDERS—REPLY OF DR. T. WILLIAMS.—In the present number of the EXAMINER will be found a letter from Dr. T. Williams, of Milwaukee, in explanation of his "Collegiate Agency," to which we had previously invited the attention of the profession in that city. Before making any comments on his letter, we will be much obliged if he will inform us what medical schools or colleges employ him to act as their agent, and sell tickets for students or practitioners who are not in attendance, and who are only expected to attend at the final examination? Also what colleges confer medical diplomas on practitioners who may have practised five years, or three years, and attended one course of lectures, without any examination (*pro forma*), and how much they charge for such diplomas? If all the operations between him, as agent, and the colleges are legitimate and honorable, as he claims, there can be no objection to giving us the additional information we ask. In the meantime, we have been informed by a correspondent, at Madison, Wisconsin, that the Legislature, now in session there, has repealed the charter of Dr. Williams "Medical Institute."

*The Arts*, is the title of a new folio sheet of 24 pages, published in this city, by Joseph M. Hirsh & Co., of this city. It is to be published monthly, and devoted to science and arts. The first number is published in good style, and it merits an extensive patronage. Price $1 per annum. Office Nos. 10 and 12 South Wells St., Chicago.

---

### PROFESSOR BIGELOW'S FINAL REPLY TO PROFESSOR GUNN.

MR. EDITOR:—The question of injustice, which is alone at issue between Professor Gunn and myself, refers to two points, viz.:

1. Did he, in opposition to the Y ligament theory, assign several different parts of the hip capsule as the essential cause of the phenomena of hip dislocation?

Unquestionably he did.

For example, he pointed out the anterior part for the dorsal, and the posterior part for the pubic dislocation.* My statement, of which he has so loudly complained, is correct.

2. In his two dorsal dislocations, did Professor Gunn, when he designated the anterior "half" of the hip capsule, point out the ilio-femoral ligament, which is a portion of this half?

Clearly, not.

He entirely fails to show that the ilio-femoral ligament even occurred to his mind, "in this relation," until my book was published. He had previously designated the capsular ligament loosely by "halves," and offers no evidence that in so doing he had analyzed or dissected the anterior half more than the posterior half.

The Y ligament theory in hip dislocations will in time pass for what it is worth. It is not the subject of the present discussion, and I therefore do not follow the new issues of Professor Gunn. If his fears of circumduction, which are shown to be unfounded, compel him to resort to a Jarvis' adjuster,† in order to reduce a thigh by manipulation, I leave to him the task of convincing the astonished reader that this is an expedient of "skill, not force."‡

The imputation of injustice, which has caused me some solicitude, is the only issue between us. I have shown that no injustice has been done to Professor Gunn, and therefore leave the discussion here.

HENRY J. BIGELOW.

* Luxations, &c., p. 20, line 20. † Ibid, pp. 13 and 14. ‡ Ibid.

---

### AMERICAN MEDICAL ASSOCIATION.

Office of Permanent Secretary, Wm. B. Atkinson, M.D.,

*1400 Pine Street, S. W. cor. Broad, Philadelphia.*

The Twenty-first Annual Session will be held in Washington, D.C., May 3, 1870, at 11 A.M.

The following Committees are expected to report:

On Cultivation of the Chincona Tree, Dr. Lemuel J. Deal, Pennsylvania, Chairman.

On the Cryptogamic Origin of Disease with special reference to recent microscopic investigations on that subject, Dr. Edward Curtis, U. S. A., Chairman.

On the Doctrine of Force, Physical and Vital, Dr. John Waters, Missouri, Chairman.

On Variola, Dr. Joseph Jones, Louisiana, Chairman.

On the Relative Advantages of Syme's and Pirogoff's mode of

Amputating at the Ankle, Dr. G. A. Otis, U. S. A., Chairman.

On a National Medical School, Dr. F. G. Smith, Pennsylvania, Chairman.

On Commissioners to aid in Trials involving Scientific Testimony, Dr. John Ordronaux, N. Y., Chairman.

On the Climatology and Epidemics of Maine, Dr. J. C. Weston; New Hampshire, Dr. P. A. Stackpole; Vermont, Dr. Henry Janes; Massachusetts, Dr. H. I. Bowditch; Rhode Island, Dr. C. W. Parsons; Connecticut, Dr. E. K. Hunt; New York, Dr. W. F. Thoms; New Jersey, Dr. Ezra M. Hunt; Pennsylvania, Dr. D. F. Condie; Maryland, Dr. O. S. Mahon; Georgia, Dr. Juriah Harriss, Missouri, Dr. Geo. Engleman; Alabama, Dr. R. F. Michel; Texas, Dr. T. J. Heard; Illinois, Dr. R. C. Hamil; Indiana, Dr. J. F. Hibberd; District of Columbia, Dr. T. Antisell; Iowa, Dr. J. C. Hughes; Michigan, Dr. Abm. Sager; Ohio, Dr. T. L. Neal; California, Dr. F. W. Hatch; Tennessee, Dr. B. W. Avent; West Virginia, Dr. E. A. Hildreth; Minnesota, Dr. Samuel Willey; Virginia, Dr. W. O. Owen; Delaware, Dr. L. B. Bush; Arkansas, Dr. G. W. Lawrence; Mississippi, Dr. W. Compton; Louisiana, Dr. L. T. Pimm; Wisconsin, Dr. J. K. Bartlett; Kentucky, Dr. J. D. Jackson.

On Veterinary Colleges, Dr. Thomas Antisell, D. C., Chairman.

On Medical Ethics, Dr. Lewis A. Sayre, N. Y., Chairman.

On American Medical Necrology, Dr. C. C. Cox, Maryland, Chairman.

To Memorialize State Medical Societies, Dr. N. S. Davis, Illinois, Chairman.

On Nomenclature of Diseases, Dr. F. G. Smith, Pennsylvania, Chairman.

On Medical Education, Dr. T. G. Richardson, Louisiana, Chairman.

On Medical Literature, Dr. J. J. Woodward, U. S. A. Chairman.

On Prize Essays, Dr. Grafton Tyler, D. C., Chairman.

Voluntary communications will be presented by—

Dr. John Curwen, Pennsylvania, on the Proper Treatment of the Insane.

Dr. Nathan Allen, Massachusetts, on the Physiological Laws of Human Increase.

Secretaries of all medical organizations are requested to forward lists of their Delegates as soon as elected, to the Permanent Secretary.

Any respectable physician who may desire to attend, but cannot do so as a delegate, may be made a *member by invitation*, upon the recommendation of the Committee of Arrangements.

W. B. ATKINSON.

---

INDIAN HEMP IN HYDROPHOBIA.—At a recent meeting (Dec. 2d) of St. Andrew's Medical Graduates' Association, a communication was read from Prof. Polli, of Milan, recommending the use of Indian hemp, in large doses, in hydrophobia. He related a case in which, although the patient died, the horror, and violence, and raving which torture hydrophobic patients were entirely subdued by its use.

In the discussion which ensued, Dr. Ross expressed an opinion that the frightful symptoms accompanying attempts to swallow were not depending on a mental or moral cause, but resulted from physical pain produced by any act of deglutition. He related a case which he had seen lately, in which Dr. Lockhart Clarke discovered no lesion of the nervous centres, but in which there was the small ulcer in the back of the pharynx, which had been described as an accompaniment of the disease.

Prof. Polli also added a note on the fact that coffee, tea, and cocoa assist, while lemon juice, citric, malic, acetic, and tartaric acids prevent, the action of Indian hemp. The latter may truly be called antidotes.—*Lancet*, Dec. 11, 1869.

---

EXUBERANCE OF LIFE IN GREAT VARIETIES AT ENORMOUS OCEAN DEPTHS.—One of the most important scientific communications received of late years by any learned society is the report on the recent deep-sea explorations, made to the Royal Society, on Thursday, Nov. 18th, by Dr. Carpenter. Our space will not allow us to make an analysis of it, but we may mention one or two of the startling facts which the deep-sea dredging has brought to light. The explorations have been conducted on board H. M. S. *Porcupine*, by Dr. Carpenter, Prof. Wyville Thompson, and Mr. Gwyn Jeffreys. In the first place, this and former explorations have fully proved, contrary to all preconceived opinions, that life exists in wonderful variety and exuberance at enormous ocean depths, and that the temperature of the deep sea presents the most remarkable variations in degree.

"More remarkable still, it was found that a difference in bottom temperature, between 32° and 47°, existed at points only eight or ten miles distant from each other, beneath a uniform surface temperature of about 52°, and that where this was the case, in the cold area, the bottom was formed of barren sandstone, mingled with fragments of older rock, and inhabited by a comparatively scanty fauna, of an artic or boreal character, while in the adjacent warm area, the bottom surface was cretaceous, and the more abundant fauna presented characteristics due to the more temperate climate."

It is easy to see how this discovery gives to the winds many of our cherished ideas, with regard to the succession of strata and of geological periods.

"The upheaval of a few miles of sea-bottom, subject to these conditions, would present to the geologist of the future two portions of surface totally different in their structure; the one exhibiting traces of a depressed, the other of an elevated, temperature; and yet these formations would have been contemporaneous and conterminous. Wherever similar conditions are found upon the dry land of the present day, it has been supposed that the high and the low temperature, the formation of chalk and the formation of sandstone, must have been separated from each other by long periods; and the discovery that they may actually coëxist upon adjacent surfaces, has done no less than strike at the very root of many of the customary assumptions with regard to geological time."

Well might Sir Charles Lyell call these discoveries almost revolutionary in their character. It has been hitherto the custom to talk and write about the dark caves and recesses of ocean, but all this must be expunged from scientific writing and left to the poets. From the most profound depths—more than 2000 fathoms, nearly the height of Mont Blanc—animals of high organization, *and with perfect eyes*, have been brought to the surface by the dredge. Sir Charles Lyell suggested that the light which these creatures evidently enjoy must be a phosphorescent one. Over the whole of the warmer areas explored, the bottom was found to be covered with globigerina deposit—animals actively engaged in chalk formation. In the colder areas these are not found, but there we have beds of volcanic sand, with whole nations of echinoderms. Besides, from these great depths the dredge has brought up delicious sponges and foraminifera, zoophytes, molluscs, annelids, and crustaceans. 127 species of molluscs, not previously known to exist in the British seas were made captive, and many of them belong to new species. Dr. Carpenter promises shortly to exhibit to the Fellows of the Royal Society, specimens of all the treasures of the deep which are thus wonderfully brought to light.—*Med. Times and Gazette.*

The Oldest Relic of Humanity.—The oldest relic of humanity extant is the skeleton of one of the earlier Pharaohs, encased in its original burial robes, and wonderfully perfect considering its age, which was deposited about 18 or 20 months ago in the British Museum, and is justly considered the most valuable of its archæological treasures. The lid of the coffin which contained the royal mummy was inscribed with the name of its occupant, Pharaoh Mikerinus, who succeeded the heir of the builder of the Great Pyramid, about ten centuries before Christ. The monarch whose crumbling bones and leathery integuments are now exciting the wonder-gazers in London, reigned in Egypt before Solomon was born, and only about 11 centures or so after Mizraim, the grandson of father Noah, and the first of the Pharaohs, had been gathered to his fathers. The tidemark of the deluge would scarcely have been obliterated when this man of the early world lived, moved, and had his being.—*Medical Record.*

MORTALITY FOR THE MONTH OF JANUARY, 1870:—

| | |
|---|---|
| Accident, burned by gasoline and naphtha | 3 |
| " asphyxia | 2 |
| " run over by wagon | 1 |
| " railroad | 1 |
| " scalded | 3 |
| " suffocation by falling in ditch | 1 |
| Abscess, hepatic | 1 |
| " of pylorus | 1 |
| Aneurism, cardiac | 1 |
| Anus, imperforate | 1 |
| Angina | 1 |
| Apoplexy | 5 |
| Ascitis | 2 |
| Asthma | 2 |
| Asphyxia | 2 |
| Atelectasis pulmonem and ulcer of abdomen | 1 |
| Births, premature | 12 |
| " still | 41 |
| Bowels, congestion and dysentery | 1 |
| Brain, congestion of | 2 |
| " & lungs, chronic disease of | 1 |
| " disease of | 1 |
| " inflammation & insanity | 1 |
| " inflammation | 6 |
| " paralysis of | 2 |
| " softening of | 2 |
| Bronchitis | 3 |
| " capillary | 6 |
| Cancer of breast | 1 |
| " of bladder and ulceration | 1 |
| Cachexia Africana | 1 |
| Cellulitis, pelvic | 1 |
| Childbirth | 2 |
| Cholera infantum and convulsions | 1 |
| Convulsions | 56 |
| " puerperal | 1 |
| Consumption | 43 |
| Croup | 27 |
| " membranous | 5 |
| Debility | 3 |
| " general | 3 |
| " and old age | 2 |
| Delirium tremens | 2 |
| Diabetes | 1 |
| Diarrhœa | 2 |
| Diphtheria | 15 |
| Dropsy | 5 |
| " general | 2 |
| Dysentery | 4 |
| " chronic, and abscess hepatic | 1 |
| Empyema | 1 |
| Encephalitis | 1 |
| Endo-carditis, rheumatic | 1 |
| Enteritis | 6 |
| " and pneumonia | 1 |
| Erysipelas | 2 |
| Fever, congestive | 1 |
| " intermittent | 1 |
| " puerperal | 4 |
| " scarlet | 49 |
| " " complications | 8 |
| " " malignant | 6 |
| " typhoid | 6 |
| Gangrene and traumatic delirium | 1 |
| Gastritis | 2 |
| Gastro-enteritis | 1 |
| Heart, disease of | 5 |
| " hypertrophy of | 1 |
| " neuralgia of | 1 |
| " valvular disease | 3 |
| " fatty degeneration of | 2 |
| Hepatitis | 1 |
| Hernia, femoral | 1 |
| Hip, disease of | 1 |
| Hydrothorax | 1 |
| Hydrocephalus | 11 |
| Inanition | 9 |
| Intemperance | 1 |
| Intestines, constriction | 1 |
| Kidneys, Bright's disease of | 2 |
| " disease of | 1 |
| " " dropsy | 1 |
| Larynx, inflammation | 1 |
| Laryngitis | 1 |
| Lungs, congestion of | 3 |
| " stomach " | 1 |
| " Paralysis of | 2 |
| Manslaughter | 1 |
| Measles | 5 |
| Meningitis, rheumatic | 1 |
| " tubercular | 1 |
| " and convulsions | 1 |
| Metro-peritonitis | 1 |
| Meningitis | 12 |
| " cerebro-spinal | 2 |
| Metritis and dysentery | 1 |
| Old age | 6 |
| Œsophagus and tracha tumor | 1 |
| " inflammation of | 1 |
| Paralysis | 7 |
| Pericarditis | 1 |
| Peritonitis | 1 |
| " puerperal | 2 |
| Pneumonia | 29 |
| " & complications | 2 |
| Pregnancy, extra uter. | 2 |
| Purpuria hemorrhagia | 1 |
| Pyæmia | 1 |
| " following amputation of forearm | 1 |
| Rheumatism | 1 |
| Scrofula | 2 |
| Small-pox | 1 |
| Spina bifida | 1 |
| Spine, inflammation of | 1 |
| Stomach, cancer of | 1 |
| Suicide, poison | 4 |
| " hanging | 2 |
| Tabes mesenterica, | 6 |
| Teething | 4 |
| Uterus, cancer of | 1 |
| Urina organs, cancer | 1 |
| Whooping-cough | 4 |
| Total | 482 |

NATIVITY.

| | |
|---|---|
| Bohemia | 2 |
| Canada | 3 |
| Chicago, Native | 95 |
| Chicago, Foreign | 157 |
| U. S., other parts | 85 |
| England | 12 |
| France | 3 |
| Germany | 51 |
| Holland | 3 |
| Italy | 2 |
| Ireland | 40 |
| Norway | 3 |
| Poland | 3 |
| Prince Edw'd Island | 1 |
| Russia | 1 |
| Sweden, | 11 |
| Scotland, | 7 |
| Switzerland | 1 |
| Unknown, | 2 |
| Total, | 482 |

AGES.

| | | | | | |
|---|---|---|---|---|---|
| Under 1 | 136 | 10 to 20 | 15 | 60 to 70 | 25 |
| 1 to 2 | 49 | 20 to 30 | 33 | 70 to 80 | 8 |
| 2 to 3 | 42 | 30 to 40 | 43 | 80 to 90 | 7 |
| 3 to 4 | 21 | 40 to 50 | 28 | | |
| 4 to 5 | 18 | 50 to 60 | 19 | Total, | 482 |
| 5 to 10 | 38 | | | | |

COMPARISON.

| | | | | | |
|---|---|---|---|---|---|
| Deaths in Jan'y, 1870, | 482 | Deaths in Jan'y 1869, | 383 | Increase, | 99 |
| Deaths in Dec., 1869, | 438 | Increase, | 44 | | |

| | | | | | |
|---|---|---|---|---|---|
| Males, | 244 | Females, | 238 | Total, | 482 |
| Single, | 368 | Married | 114 | Total, | 482 |
| White, | 478 | Colored, | 4 | Total, | 482 |

MORTALITY BY WARDS FOR THE MONTH.

| Wards. | Mortality |
|---|---|
| 1 | 8 |
| 2 | 17 |
| 3 | 20 |
| 4 | 12 |
| 5 | 27 |
| 6 | 26 |
| 7 | 21 |
| 8 | 36 |
| 9 | 36 |
| 10 | 16 |
| 11 | 15 |
| 12 | 16 |
| 13 | 9 |
| 14 | 9 |
| 15 | 47 |
| 16 | 26 |
| 17 | 24 |
| 18 | 29 |
| 19 | 21 |
| 20 | 25 |

| | Mortality. |
|---|---|
| Accidents | 11 |
| County Hospital | 7 |
| Home for Friendless | 2 |
| Hospital for Women and Children | 1 |
| Immigrants | 1 |
| Jewish Hospital | 2 |
| Manslaughter | 1 |
| Mercy Hospital, | 4 |
| Protestant Orphan Asylum | 1 |
| St. Luke's Hospital | 1 |
| St. Joseph Orphan Asylum, | 5 |
| Suicides | 6 |
| Total, | 482 |

